# Current Landscape of Molecular Biomarkers in Gastroesophageal Tumors and Potential Strategies for Co-Expression Patterns

**DOI:** 10.3390/cancers17030340

**Published:** 2025-01-21

**Authors:** Martin Korpan, Hannah Christina Puhr, Julia M. Berger, Alexander Friedrich, Gerald W. Prager, Matthias Preusser, Aysegül Ilhan-Mutlu

**Affiliations:** 1 Division of Oncology, Department of Medicine I, Medical University of Vienna, Waehringer Guertel 18-20, 1090 Vienna, Austria; 2 Christian Doppler Laboratory for Personalized Immunotherapy, Department of Medicine I, Medical University of Vienna, Waehringer Guertel 18-20, 1090 Vienna, Austria

**Keywords:** gastric cancer, esophageal cancer, gastroesophageal tumors, molecular biomarkers, HER-2, PD-L1, microsatellite instability, CLDN18.2, TIGIT, DKK-1

## Abstract

The treatment of metastasized gastroesophageal adenocarcinoma largely depends on molecular profiling based on immunohistochemical procedures. Therefore, the examination of HER2, PD-L1, and dMMR/MSI is recommended by the majority of clinical practice guidelines, as positive expression leads to different treatment approaches. Data from large phase-III trials and consequent approvals in various countries enable physicians to offer their patients several therapy options including immunotherapy, targeted therapy, or both combined with chemotherapy. The introduction of novel therapeutic targets such as CLDN18.2 leads to a more complex decision-making process as a significant number of patients show positive results for the co-expression of other biomarkers besides CLDN18.2. The aim of this review is to summarize the current biomarker landscape of patients with metastatic gastroesophageal tumors, its direct clinical impact on daily decision-making, and to evaluate current findings on biomarker co-expression. Furthermore, possible treatment strategies with multiple biomarker expression are discussed.

## Simple Summary

Gastroesophageal cancer is still a disease with poor survival, particularly in metastatic settings. Personalized therapy approaches rely on the expression of molecular markers in each individual tumor. Novel treatment regimens targeting these molecular markers are underway to improve patient management and outcome. In recent years, the focus has shifted to therapeutic approaches addressing biomarker co-expression and further advancements are anticipated in the future. This review aims to highlight the current and future biomarker landscape combined with clinical trials in metastasized gastroesophageal adenocarcinoma and summarizes the therapeutic strategies for multiple target expression.

## 1. Introduction

Gastroesophageal tumors (GETs), which comprise esophageal cancer (EC), gastric cancer (GC), as well as cancer of the gastroesophageal junction (GEJ), are a common disease and significant contributor to global disease burden. With about 1.4 million newly diagnosed patients in 2022, the disease frequently presents in an advanced stage where surgical resection is limited [1]. Incidence rates differ geographically, with the highest age-standardized rate (ASR) documented in Eastern Asia [1]. In 2022, the ASR per 100,000 for GET among both sexes was 17.3 in Asia and 11.2 in Europe [2]. Although incidence levels for GC steadily decline, an increased risk has been observed in younger generations (aged < 50 years) [3]. This mainly concerns high-income countries such as the United Kingdom and the United States, which might be linked to elevated obesity rates [3].

Despite an improved survival rate across all tumor stages in recent decades, the prognosis of GET remains poor, particularly in metastatic settings. The five-year relative survival for GC and EC is 35.8% and 28.1% in regional and only 7.0% and 5.3% in metastatic stages, respectively [4,5]. The most common histological classifications for gastric adenocarcinoma include the Lauren and World Health Organization (WHO) classification. Based on the Lauren classification, there are the intestinal, diffuse, or mixed GC subtypes [6]. Alternatively, the WHO system groups GC into four main subtypes including tubular, papillary, poorly cohesive (including signet ring cell carcinomas), mucinous, and other rare variants [7]. The intestinal subtype by Lauren approximately corresponds to papillary and tubular carcinomas, while the diffuse subtype relates to poorly cohesive carcinomas [7]. These classifications underline the heterogeneity of this disease but are insufficient to establish precise therapy strategies for individual patients. Consequently, in recent decades, there have been substantial advances in individual patient-based treatment according to the expression of biomarkers, leading to a change in the therapeutic landscape [8]. The results of The Cancer Genome Atlas (TCGA) project proposed a classification of gastric adenocarcinoma into four molecular subtypes: Epstein–Barr virus (EBV)-positive, microsatellite unstable, genomically stable, and chromosomal instable tumors [9].

Such and further molecular biomarkers set the breakthrough in targeted treatment options and individualized patient-care, which in consequence led to intensified research. In this review, molecular biomarkers will be discussed, particularly the interaction between them in advanced gastroesophageal adenocarcinoma (GEA) and consequent multitarget therapy strategies. The majority of recommendations are derived from Western guidelines, although Asian guidelines are also mentioned where appropriate.

## 2. Current Standard Biomarkers

### 2.1. HER2

The human epidermal growth factor receptor 2 (HER2) protein (also known as ERBB2) is a transmembrane tyrosine kinase and part of the epidermal growth factor receptors (EGFRs) family [10]. HER2 acts as a key driver of tumorigenesis in various cancer types including GET, but may be found in a diverse subgroup of tumors including bladder, breast, uterine, cholangiocellular, colorectal, lung, and pancreatic cancer [11]. The overexpression of HER2 in the cell membrane or its gene amplification endows malignant cells with malignant features such as proliferation, angiogenesis, and invasion [12,13,14]. HER2 positivity is observed in approximately 15–20% of all GET [9,15]. Similar rates were observed between European and Asian patients; however, there was much higher positivity in intestinal- compared to diffuse-type tumors and in GEJ [15]. Before the development and approval of HER2 directed treatment in GET, several studies implied that HER2-positivity was associated with a poor outcome and more rapid disease progression [10,16,17]. Nowadays, due to additional treatment options, HER2 positivity is regarded as a clinically relevant targeted treatment option in patients with stage IV GET [18]. The ESMO Scale for Clinical Actionability of Molecular Targets (ESCAT) allows clinicians to prioritize targets in each individual patient according to biomarker expression [19]. According to ESMO guidelines, HER2 has the highest ESCAT score possible (I-A) [20].

Consequently, the HER2 status needs to be assessed either by fluorescent in situ hybridization (FISH) or by immunohistochemistry (IHC) in all patients with advanced GET prior to therapy initiation [20]. Since there is extensive intratumoral heterogeneity of HER2 expression in GC, multiple biopsies (i.e., 5–8) are recommended to determine the HER2 status [20,21]. As there may be a significant discrepancy between the primary tumor, lymph nodes, and metastatic tissue, several biopsies of the same location prevent potential sampling errors and guarantee the efficacy of targeted treatment [22,23,24]. Even the fixation time of the tumor tissue in formalin may have an impact on HER2 detection. A retrospective analysis demonstrated that there is a risk of underestimating the HER2 status due to the over-fixation of tissue samples which are submitted on weekends [25]. Such heterogeneity poses a huge challenge, as the comparability of tissue samples is not ensured. To address this issue, the VARIANZ study was conducted to explore discrepancies between local and central testing [26]. These discrepancies were shown to significantly impact treatment outcomes in targeted therapies [26]. Consequently, patients with different HER2 results performed by two independent laboratories had no benefit in targeted treatment with the anti-HER2 monoclonal antibody (mAb) trastuzumab. This underlines the importance of technical procedures in HER2 determination. In line with this, the exact thresholds of HER2 expression are defined, as borderline HER2 positivity should be considered a resistance factor for targeted treatment [26].

The phase-III ToGA trial demonstrated the benefit of the anti-HER2 mAb trastuzumab in combination with platinum-fluoropyrimidine chemotherapy (CHT) in patients with advanced GC, especially in a subgroup with HER-2 positivity, which was identified later on in a post hoc analysis as a HER2 IHC score 3+ alone or HER2 IHC 2+ and FISH positivity [15,27]. In the primary analysis group containing patients with IHC ≥ 1 or gene amplification, the addition of trastuzumab resulted in a significant improvement in overall survival (OS, 13.8 versus 11.1 months), progression-free survival (PFS, 6.7 versus 5.5 months), and response rate (47% vs. 35%) [27].

On the other hand, several different anti-HER2 targeted agents failed to improve survival for GET patients. Lapatinib, a small molecule dual EGFR and HER2 tyrosine-kinase inhibitor, which is already approved for HER2-positive metastatic breast cancer, showed promising in vitro and in vivo results, but did not succeed in clinical trials as first-line therapy for metastatic GET [28,29]. In addition to capecitabine and oxaliplatin, lapatinib did not lead to a significantly higher OS and PFS, but only to a significantly higher response rate (53% vs. 39%) in the large phase III TRIO-013/LOGiC trial [30]. Furthermore, a dual targeted approach consisting of pertuzumab + trastuzumab + cisplatin + capecitabine or 5-fluouracil (5-FU) showed no significant survival benefit compared to trastuzumab + CHT alone in patients with untreated metastatic GET [31]. However, the end-of-study analyses confirmed some agent activity, as the median OS after 44.4 months’ follow up was 18.1 months in the pertuzumab arm and 14.2 months in the placebo arm, which still did not meet the predefined statistical significance threshold (stratified hazard ratio (HR) 0.85, 95% confidence interval (CI) 0.72–0.99, *p* = 0.057) [32]. A subgroup-analysis focusing on Japanese and Chinese patients showed similar efficacy to that in the overall population [33,34].

Anti-HER2 directed therapy was tested in diverse trials as second line approaches after progression to trastuzumab-containing treatment. The Asian TyTAN trial, where lapatinib + paclitaxel was compared to paclitaxel alone, did not meet the primary endpoint of prolonged OS [35]. Analogously to the LOGiC trial, the addition of lapatinib led only to an increased response rate in the intention-to-treat (ITT) population (27% vs. 9%, *p* = 0.001) [35].

Similarly, the antibody–drug conjugate (ADC) trastuzumab emtansine (T-DM1) was not superior to CHT (taxane) in pretreated metastatic GET patients [36]. In this phase-III GATSBY trial, patients who received T-DM1 had a median OS of 7.9 months (95% CI 6.7–9.5 months), while the control arm with CHT had a median OS of 8.6 months (HR 1.15, 95% CI 0.87–1.51, one-sided *p* = 0.86) [36].

However, trastuzumab deruxtecan (T-DXd), another ADC shows considerable advantages when compared to T-DM1. T-DXd consists of an innovative topoisomerase I inhibitor, which is connected via an enzymatically cleavable peptide linker to trastuzumab [37]. First, the drug-to-antibody ratio is much higher in T-DXd, leading to an enhanced cytotoxic potential. In addition, the so-called bystander killing effect was observed, meaning that T-DXd kills both HER2-positive cells and adjacent HER-2 negative cells [38]. This is particularly crucial in terms of the already mentioned intratumoral HER2 heterogeneity and might explain the failing of T-DM1 [38]. Its benefit was first observed in the Asian phase-II DESTINY-Gastric01 trial in pretreated HER2-positive advanced GC patients, while the control group received CHT (median OS 12.5 vs. 8.4 months, HR 0.59, 95% CI 0.39–0.88, *p* = 0.01) [39]. Patients with IHC 3+ showed greater benefit than those with IHC 2+ and FISH positivity. The promising antitumor effects in a Western population were confirmed in the subsequent single-arm phase-II DESTINY-Gastric02 trial, where patients received T-DXd after progressing on a first-line therapy containing trastuzumab [40]. A confirmed overall response rate (cORR) was observed in 33 out of 79 patients (42%), including four complete responses (CRs) [40]. Even in heavily pretreated GEA patients with no prior anti-HER2 therapy and a low HER2 score (IHC 2+ and FISH negative or IHC 1+), T-DXd showed antitumor activity, which underlines the potency of this ADC [41]. Although this new classification is already implemented in the treatment armamentarium of metastatic breast cancer, information on daily clinical use in GET is not mature [42]. Different anti-HER2 strategies are now currently being tested in patients with a low Her2 score [43].

These results need to be validated in larger cohorts as well as in combination with other drug agents, which led to the development of the following trials: the phase-III DESTINY-Gastric04 trial (NCT04704934), comparing second-line T-DXd to the standard of care ramucirumab + paclitaxel in a large GEA cohort and the phase-Ib/II DESTINY-Gastric03 trial (NCT04379596), which aims to assess the safety and efficacy of T-DXd with different combinations including immunotherapy, CHT or both in advanced HER2 expressing GC [44,45]. Due to the need to perform extensive research for HER2-positive GET, a broad range of novel treatment strategies is currently being investigated. These include a new anti-HER2 mAb (margetuximab), another tyrosine kinase inhibitor (tucatinib), bispecific antibodies (zanidatamab and KN026), ADC (disitamab vedotin), and a vaccine, i.e., B-cell immunotherapy-based on HER-2 peptides [46,47,48,49,50,51].

As predicting the outcome of targeted HER2 therapy remains a challenge, extrachromosomal DNA (ecDNA) might play an important role as a prognostic factor for improved survival in anti-HER2-treated GET patients. In an experimental analysis comprising 162 patients treated with first-line HER2 inhibition, ERBB2-amplified ecDNA+ (*n* = 77) was found to be associated with prolonged PFS and OS compared to ERBB2 non-amplified ecDNA- (PFS HR 2.60, 95% CI 1.70–3.98, *p* = 0.02; OS HR 3.42, 95% CI 2.18–5.36, *p* < 0.0001) [52].

In conclusion, there is still a huge need to find the most effective targeted therapy approach for HER2-positive GET, which necessitates further research and clinical trials.

### 2.2. Programmed Death Protein 1 (PD1)

Several clinical trials have already demonstrated the benefit of immunotherapy such as PD-1 inhibitors in GET. To determine the eligibility for applying immune-checkpoint inhibitors (ICIs), the immunohistochemical staining of programmed death-ligand 1 (PD-L1) should be performed in locally advanced or metastatic GET [20]. A study performed to assess the most accurate PD-L1 score for the entire tumor based on biopsy analysis concluded that obtaining four surface biopsies provided the most precise results [53].

**Figure 1:**
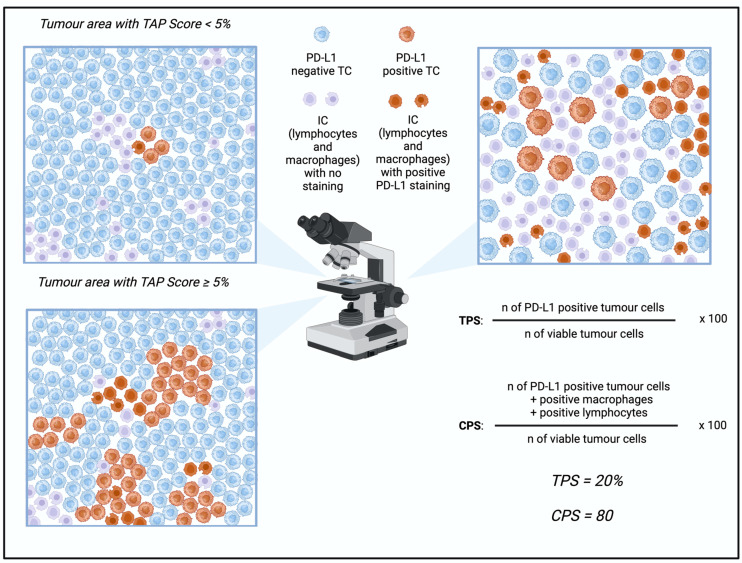
Comparison of the TAP to TPS and CPS. The visual estimation of tumor area positivity is less time consuming than counting each cell. CPS = combined positive score; IC = immune cell; *n*, number; PD-L1 = programmed death-ligand 1; TAP = tumor area positivity; TC = tumor cell; TPS = tumor proportion score.

Although there are several scores available to assess the PD-L1 expression, the combined positive score (CPS) is mainly used in GEA [20]. In contrast to the tumor proportion score (TPS), which evaluates viable tumor cells with either partial or complete membrane staining at any intensity, the CPS also includes lymphocytes and macrophages [54]. This means that CPS is the entire number of PD-L1 staining cells (tumor cells, lymphocytes, macrophages) divided by the total quantity of viable tumor cells, multiplied by 100 [54]. Although different cut-offs exist, a CPS cut-off of ≥ 1 indicates a positive PD-L1 expression [20,55]. An additional score is the tumor area positivity (TAP), which is determined by visually estimating the ratio of PD-L1 positive tumor cells and tumor-associated immune cells to the total tumor area [56]. When comparing the TAP to the CPS score, a study concluded that both scoring systems are of similar effectiveness, but using TAP is significantly less time-intensive [57]. The average time a pathologist needed for scoring TAP was 5 min, whereas it was 30 min for the CPS, underlining the time-consuming procedure of counting cells [57]. In line with this, a PD-L1 subgroup analysis from the phase-III RATIONALE-305 trial observed that TAP and CPS displayed significant concordance, with an interclass correlation coefficient of 0.81 (95% CI 0.79–0.83) [58]. This suggests that both methods are similarly eligible for the clinical assessment of PD-L1 expression, but in terms of time efficiency, the TAP scoring method might be more advantageous. An overview of the different scoring methods is shown in Figure 1.

Since there are various antibodies utilized for PD-L1 staining in GET, the question of interchangeability arose. Therefore, PD-L1 22C3 and 28–8 pharmDx assays, which had been used in large phase III studies, were compared at different CPS cut-offs (1, 10, and 50) in 55 gastric AC tissue samples [59]. This study concluded that both antibodies have a concordance rate of 96.4%, 96.4%, and 100% for each CPS cut-off, respectively, and thus may be seen as interchangeable [59]. On the other hand, a more recent study examining 362 GC specimens contradicts this assumption of interchangeability. It suggests that assessing the PD-L1 CPS with the 28–8 assay may lead to increased PD-L1 scores and an increased proportion of PD-L1 positivity in comparison to 22C3 and other assays [60]. Consequently, equivalence between these two staining antibodies and, hence, interchangeability, is still not fully proven, which necessitates further studies on inter-assay similarity.

The ASPIRE study aims to provide a definitive answer to the interchangeability of different scoring systems (mainly TAP vs. CPS) and the usage of various staining antibodies [61]. Consequently, a conclusion on this topic and the establishment of standardization is soon to be expected.

The results of the Checkmate 649 trial led to the approval of the anti-PD-1 inhibitor nivolumab + CHT in standard of care (SOC) first-line therapy setting for PD-L1 positive (CPS ≥ 5) advanced/metastatic unresectable GEA: Both primary endpoints, i.e., OS and PFS, were met and significantly improved in the nivolumab cohort [62]. Data from the 3- and 4-year follow-up of Checkmate 649 confirmed previous findings, showing improvements in OS, PFS, and the objective response rate (ORR) across all patients [63,64]. The biggest benefit was observed in patients with CPS ≥ 5 [63,64].

Another meaningful observation was that the response at the 18-week landmark may be useful to predict OS [63]. There was an increased number of this specific patient cohort alive at 36 months than non-responders in PD-L1 CPS ≥ 5, <5, and among the overall population [63]. However, the second experimental arm, i.e., nivolumab + the CTLA-4 inhibitor ipilimumab, was closed due to futility [64]. This evidence consequently points out the necessity of a CHT backbone in this patient cohort.

A further approved first-line treatment approach for patients with advanced GET is the PD-1 inhibitor pembrolizumab combined with CHT (capecitabine + oxaliplatin or fluorouracil + cisplatin). The KEYNOTE-859 trial demonstrated its superiority over placebo + CHT in terms of OS, PFS, and ORR, regardless of PD-L1 expression [65]. However, larger effects were observed in PD-L1 CPS-positive subgroups [66].

The American National Comprehensive Cancer Network (NCCN) guidelines already mentioned both nivolumab and pembrolizumab + fluoropyrimidine (FP, fluorouracil or capecitabine) + oxaliplatin/cisplatin for unresectable locally advanced, recurrent or metastatic GET, but with different cut-off values [55]. For category 1, pembrolizumab may be administered for PD-L1 CPS ≥ 10, whereas nivolumab for PD-L1 CPS ≥ 5 GET. For category 2, these ICI may be considered for lower CPS values. In parallel, the European Society for Medical Oncology (ESMO) guidelines recommend the administration of immunotherapy (either nivolumab or pembrolizumab) after a cut-off value of ≥ 5, while immunotherapy was mentioned as “could be considered” for patients having a CPS 1–4, underlining the fact that the expected extent of benefit from immunotherapy for those patients is low [20]. The European Medicines Agency (EMA), however, already approved pembrolizumab for CPS ≥ 1 [67].

Moreover, the phase-III RATIONALE 305 trial demonstrated that combining the PD-1 inhibitor tislelizumab with CHT significantly and meaningfully improved OS compared to CHT alone, with acceptable safety in 546 participants with PD-L1–positive GEA [68]. This trial used the TAP score with a cut-off of ≥ 5 for PD-L1 positivity and recently obtained a positive opinion for tislelizumab with this TAP threshold value from the Committee for Medicinal Products for Human Use (CHMP) of the EMA [69]. Results from the 3-year follow-up showed the overall superiority of tislelizumab. Especially worth noting was the PFS at 36 months of 15% in the tislelizumab group vs. 7.5% in the control group [70]. By contrast, the discontinuation rate due to adverse events (AE) was more than two times higher in the tislelizumab group (16.7% vs. 8.1%) [70].

As predicting ICI treatment outcomes remains a challenge, artificial intelligence (AI)-based digital pathology might help overcome this obstacle in the future. A single-cell computational pathology method identified 66 biomarkers, concluding that the characterization of neutrophile and lymphocyte interaction provided the highest association with PFS (HR = 0.44, 95% CI 0.24–0.79, *p* = 0.0046) [71]. Based on hematoxylin and eosin images, this study applied deep learning for tumor area detection and automated segmentation. This process enabled the classification of cell nuclei into four cell types: tumor cells, lymphocytes, neutrophils, and macrophages, allowing for a detailed analysis of the tumor microenvironment [71]. Another approach is the use of circulating tumor DNA (ctDNA) to predict response. In a small cohort of 25 patients, a reduction in ctDNA levels before first restaging or ctDNA clearance after seven cycles of pembrolizumab treatment were statistically significant predictors of PFS and OS (HR = 0.52 for PFS, *p* = 0.012, HR = 0.15 for OS, *p* = 0.005) [72]. This is in line with another study assessing 46 patients treated with anti-PD ICI, observing that the median PFS was 7.4 months for patients with undetectable post-treatment ctDNA vs. 4.9 months for those with detectable ctDNA (*p* = 0.025) [73].

In conclusion, determining the PD-L1 status is indispensable in advanced GET, as positivity offers immunotherapy administration and marks a meaningful predictive biomarker.

### 2.3. Microsatellite Instability (MSI) and Mismatch–Repair Deficiency (dMMR)

GET can be classified in MSI–low (MSI-L), microsatellite stable (MSS), or microsatellite instability–high (MSI-H). Research indicates that MSI-H status may serve as a predictive biomarker in advanced GET, as MSI-H and mismatch–repair deficiency (dMMR) are linked to an elevated response rate and favorable survival outcome after the administration of immunotherapy [74,75,76]. In advanced GEA, MSI-high has the highest predictive value for ICI response, even before high PD-L1 CPS values such as CPS ≥10 [77]. Subsequently, MMR testing with IHC should be performed for all newly diagnosed, advanced, unresectable, and stage IV GET [20,55]. If one or more MMR proteins no longer show a nuclear expression (=dMMR), the likelihood of MSI-H is increased. In this case, mismatch DNA is no longer repaired, which results in genomic mutation and the development of immunogenic tumor-specific antigens (=neoantigens), which can be identified by T-cells [78]. According to the ESCAT of the ESMO GC living guidelines, dMMR/MSI-H is also classified as I-A, underlining the importance of this biomarker [79].

In the KEYNOTE-059 trial, tissue MSI status was available in 174/259 previously treated GET patients who received pembrolizumab [80]. MSI-H was detected in seven patients, four of whom had an ORR (57.1%; 95% CI, 18.4–90.1%). Among 167 patients with non–MSI-high specimens, 15 experienced ORR (9.0%; 95% CI, 5.1–14.4%) [80]. Although the primary endpoint of the KEYNOTE-061 trial, i.e., prolonged OS, was not met in patients with PD-L1 CPS of ≥1, pembrolizumab alone led to better response and longer OS in contrast to paclitaxel monotherapy in MSI-H GET patients [81]. In the MSI-H subgroup of the KEYNOTE-062 trial, patients treated with pembrolizumab ± CHT had longer OS, PFS and response duration compared to patients treated with CHT solely and to patients with non-MSI tumors treated with pembrolizumab and CHT [82]. The greatest OS benefit in the 3-year follow-up of Checkmate 649 was seen in MSI-H patients treated with nivolumab + CHT with a median OS of 44.8 months compared to 14.3 months in the MSS group (HR 0.29, 95% CI 0.12–0.71) [63]. As a result, MSI–high GET patients should definitely be considered to be treated with ICI ± CHT. The question of whether it is possible to omit CHT in this patient cohort was faced in the Japanese NO LIMIT study. Out of 935 screened cases of advanced MSI-high GET, 29 patients were enrolled and received nivolumab + ipilimumab as first-line treatment [83]. ORR was observed in 62.1% of patients, the disease control rate (DCR) even reached 79.3% and the 12-month PFS and OS rates were 73% and 80%, respectively [84]. However, due to the short follow-up period, mature data on survival outcome are lacking.

### 2.4. Tumor Mutational Burden (TMB)

The significance of TMB in GET is yet to be determined. Although the NCCN guidelines include TMB testing for GET, ESMO guidelines still do not currently mention it [20,55]. In an exploratory analysis of the KEYNOTE-061 trial, tissue TMB was found to be positively associated with clinical outcomes with pembrolizumab (ORR, *p* < 0.001; PFS, *p* < 0.001; OS, *p* = 0.003), but not with paclitaxel treatment (ORR, *p* = 0.047; PFS, *p* = 0.605; OS, *p* = 0.084) [85]. Among patients with tissue TMB ≥10 mutations/megabase (mut/Mb), pembrolizumab improved OS vs. paclitaxel, a benefit that persisted when patients with MSI-H status were excluded [85]. The same analysis was conducted for KEYNOTE-062, which confirmed the previous results: TMB showed a significant correlation with clinical outcomes in patients treated with pembrolizumab and the pembrolizumab + CHT combination (ORR, PFS, and OS; all *p* < 0.05), but not with CHT alone (all *p* > 0.05) [86]. Out of 25 MSI-H patients in KEYNOTE-062, 22 (88%) had TMB ≥ 10 mut/Mb, whereas there were 28 (10%) patients in the MSS subgroup (*n* = 281). Unfortunately, no data on the interlink between these two parameters were published for KEYNOTE-061. In the non–MSI-H subgroup analysis, the correlation between clinical outcomes (with pembrolizumab or pembrolizumab + chemotherapy) and tumor mutational burden (TMB) as a continuous variable was reduced [86]. Similarly, the clinical benefit of pembrolizumab ± chemotherapy over chemotherapy alone, based on a TMB cutoff, was less pronounced in this subgroup [86]. Another recent study concluded that TMB may serve as predictive factor for the treatment response of ICI in advanced GC [87]. TMB-high (≥14.31 mut/Mb) patients had a better ORR and OS compared to TMB-low patients treated with pembrolizumab or nivolumab. However, it was not reported whether this benefit remained when MSI-H patients were to be excluded. The FDA approved the use of pembrolizumab in TMB-high solid tumor patients, irrespective of the tumor type, using a cut-off of 10 mut/Mb. This was due to the KEYNOTE-158 results [88].

Overall, there is a strong link between TMB and MSI, which consequently leaves the sole position of TMB still to be resolved.

### 2.5. Claudin 18.2

**Figure 2:**
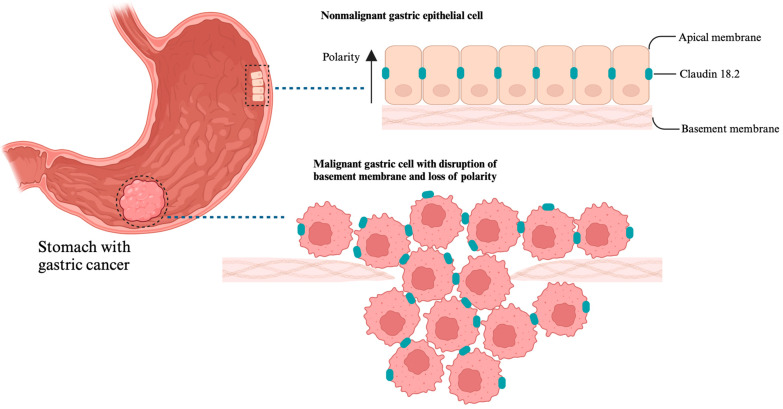
During the process of carcinogenesis, the loss of cellular polarity leads to aberrant Claudin 18.2 expression, which is associated with irregular proliferation and invasion.

As a vital component of tight junction proteins, Claudin 18 isoform 2 (CLDN18.2) is part of the claudin family and plays a crucial role in regulating tissue permeability, signal transduction and paracellular transport [89]. In normal tissue, CLDN18.2 is expressed solely on the epithelial cells of the gastric mucosa and this expression remains upon malignant transformation [90]. The disruption of the tight-junction structure due to abnormal claudin expression impairs cellular polarity and differentiation. Such pathological tight-junction structure may foster invasion and potentially facilitate irregular proliferation and induce carcinogenesis (Figure 2) [91]. The shift in polarity between nonmalignant and tumor cells, resulting in the redistribution of CLDN18.2 from tight junctions to cell membranes, may contribute to the specificity and efficacy of anti-CLDN18.2 therapies.

This molecule is considered to be a pan-cancer target, as an orthotopic expression was found in various cancer types, such as in gastric, esophageal, pancreatic, ovarian, and lung tumors (ESCAT-score I-A) [79,90]. The predominance of CLDN18.2 expression according to the histological subtype remains controversial. Some studies suggested a statistical significance with diffuse-type GC [92,93,94], while other studies and a meta-analysis concluded no association with the Lauren classification [95,96]. However, in terms of anatomical prevalence, GEJ tumors seem to express CLDN18.2 positivity more rarely than GC [96].

Although different cut-offs for CLDN18.2 positivity have been assessed, consensus was reached upon ≥75% of tumor cells expressing membranous CLDN18.2 staining and this is found in about 24–38% of GET (ESCAT I-A) [79,96,97,98]. Similarly to HER2 evaluation, there might be some heterogeneity regarding the CLDN18.2 expression patterns [99]. In a retrospective study with peritoneally disseminated GC, there were instances where CLDN18 positivity differed between biopsy and surgical specimens, with some biopsies showing negative results while the matched surgical specimens were positive for CLDN18.2 [99]. Hence, another study suggests at least six biopsies to evaluate CLDN18.2 expression [100]. No discrepancy could be observed when staining CLDN18/CLDN18.2 (*n* = 91 tumors) with different IHC assays, thus showing coherent analytical outcomes [101].

Several clinical trials have been performed using the mAb zolbetuximab in CLDN18.2-positive, HER2-negative, locally advanced unresectable, or metastatic GEA (mGEA). Starting with the single-arm phase-II MONO trial, monotherapy with the anti-CLDN18.2 mAb zolbetuximab was well tolerated and efficient [102]. In the consecutive phase-II FAST trial, the combination of zolbetuximab and first-line CHT (epirubicin, oxaliplatin, capecitabine) showed an overall survival (OS) advantage in patients with CLDN18.2 expression ≥ 70% [103]. The inclusion cut-off for moderate-to-strong CLDN18.2 expression in the FAST study was defined as ≥40%.

Subsequently, the phase-III SPOTLIGHT trial demonstrated that zolbetuximab + mFOLFOX6 (modified folinic acid (or levofolinate), fluorouracil and oxaliplatin regimen) led to longer OS and PFS vs. placebo + mFOLFOX6 in patients with CLDN18.2-positive, HER2-negative, locally advanced unresectable or mGEA [98]. Final survival results revealed that median PFS in the ITT group was significantly better with 11.04 months vs. 8.94 months in the placebo group (HR 0.73, 95% CI 0.59–0.95, *p* = 0.0024) [104]. Median OS in the ITT group was significantly longer too (18.23 vs. 15.57 months, HR 0.78, 95% CI 0.64- 0.95, *p* = 0.0075). ORR was similar between treatment arms in the ITT population and time to progression for patients with best overall response of complete/partial response was only numerically, but not statistically significantly longer in the zolbetuximab cohort [104].

Zolbetuximab + another CHT backbone (capecitabine + oxaliplatin (CAPOX)) was explored in the phase-III GLOW trial: Patients in the zolbetuximab group achieved a median PFS of 8.21 months vs. 6.80 months in the placebo group (HR = 0.69, 95% CI, 0.54–0.87, *p* < 0.001), whereas the median OS was 14.39 months in the zolbetuximab group vs. 12.16 months in the control group (HR = 0.77, 95% CI 0.62–0.97, *p* = 0.0118) [97]. Alongside with the US Food and Drug Administration (FDA) approval, the CHMP recommended zolbetuximab combined with FP- and platinum-containing CHT as first-line treatment of locally advanced unresectable or metastatic CLDN18.2 positive, HER2-negative gastric, or GEJ AC, enabling a more rapid final approval of the EMA [105].

Besides zolbetuximab, a different IgG1 mAb termed FG-M108, which targets CLDN18.2 and displays antibody-dependent cellular cytotoxicity, was assessed in a phase I/II study [106]. 52 Chinese patients with CLDN18.2 positive mGEA were treated with FG-M108 + CAPOX [106]. This mAb was well tolerated and led to a median PFS of 9.6 months (95% CI 6.7-NR), whereas OS data were not revealed yet.

Another therapy strategy that is currently being investigated is the use of CLDN18.2-specific chimeric antigen receptor T-cell (CAR-T) treatment in advanced GEA [107]. Early data from a phase I trial revealed promising antitumor effects with an acceptable safety profile in heavily pretreated patients with gastrointestinal tumors, allowing the enrolment of larger phase-II and -III trials [108]. Furthermore, several anti-CLDN18.2 antibody–drug conjugates have demonstrated promising clinical efficacy in phase-I trials in patients with CLDN18.2-positive GET, with pivotal phase-II and -III trials planned in the future [109,110,111].

As the CLDN18.2 expression was found to be stable over time and due to its high prevalence, testing for this biomarker should be considered a standard clinical procedure in GET [112].

## 3. Clinically Significant Co-Expression Clusters

### 3.1. PD-L1 and HER2

As trastuzumab promotes HER2-specific T cell responses and elevates PD-L1 expression in tumors, anti-PD-1 antibodies can further amplify these T cell-specific immune effects of trastuzumab, which led to the initiation of combined phase-II trials [113,114]. Pembrolizumab + trastuzumab + CHT (capecitabine and oxaliplatin) were administered in untreated HER-2-positive metastatic GET patients, irrespective of PD-L1 status [115]. In 37 patients, ORR was 81% and the median PFS was 14.2 months. No correlation between PD-L1 status and PFS or OS was observed [116]. Another phase-II trial showed similar findings, with a considerable tumor shrinkage for HER2-positive advanced GET [117]. In this trial, PD-L1 status had no influence on survival either. These promising preliminary results and acceptable drug safety led to the approval of the phase-III trial KEYNOTE-811. In the pembrolizumab + trastuzumab group (*n* = 133), 74.4% had an ORR, whereas ORR was achieved by only 51.9% in the placebo group (*n* = 131, *p* < 0.001) [118]. Additionally, responses in patients receiving pembrolizumab were deeper than those placebo (median change from baseline with ≥ 80% decrease, 32.3% vs. 14.8%) and CR were detected more often (11.3% vs. 3.1%) [118]. At the third interim analysis, median PFS was 10.0 months (95% CI 8.6–12.2) in the pembrolizumab group vs. 8.1 months (95% CI 7.1–8.6, HR 0.73, 95% CI 0.61–0.87) in the placebo group, meaning statistically significant longer PFS with pembrolizumab. Median OS was numerically longer; however, it did not reach the significance threshold set by the prespecified criteria across all patients (20.0 vs. 16.8 months, HR 0.84, 95% CI 0.70–1.01) [119]. Among the PD-L1 CPS ≥ 1 subgroup (85% of patients), OS was statistically significantly longer in the pembrolizumab cohort (20.0 months, 95% CI 17.9–22.7) compared to the placebo group (15.7 months, 95% CI 13.5–18.5, HR 0.81, 95% CI 0.67–0.98). Data from the final analysis confirmed the superiority of pembrolizumab + SOC vs. placebo + SOC [120]. Median PFS was 10.0 vs. 8.1 months (HR 0.73, 95% CI 0.61–0.87), and median OS 20.0 vs. 16.8 months (HR 0.80, 95% CI 0.67–0.94) [120]. As the ORR data already led to a conditional FDA approval of pembrolizumab + trastuzumab + fluoropyrimidine and platinum-containing CHT for the first-line treatment of locally advanced or metastatic HER2-positive GET irrespective of PD-L1 status, this approval was now restricted with the presented data to tumors with a CPS ≥ 1 [119,121]. A chemotherapy-free approach was examined in cohort A of the MAHOGANY trial, in which margetuximab, an anti-HER2 mAb, was administered with retifanlimab, an anti-PD-1 mAb, in first-line metastatic HER2-positive/PD-L1 positive (CPS ≥ 1) GEA [122]. This strategy may allow certain patients with a strong and sustained response to avoid CHT, thus preventing overtreatment in this biomarker-identified population. Although this therapy regimen met the predefined threshold for antitumor activity and presented a preferable toxicity profile relative to the past results with CHT and trastuzumab, this cohort A arm was discontinued [122]. Enrollment was terminated by the sponsor for business reasons, emphasizing that CHT continues to play a pivotal in treating GEA, while this CHT-free treatment approach showed less efficacy than anticipated [122].

HERIZON-GEA-01 was developed as a global phase-III trial evaluating the efficacy of the mAb zanidatamab + CHT ± tislelizumab vs. SOC (trastuzumab + CHT) as first-line treatment for patients with HER2-positive mGEA [123]. The CHT backbone consists of either CAPOX or 5-FU+ cisplatin and the primary endpoints will be PFS and OS. The recruitment of an estimated number of 918 patients is still ongoing.

A phase-Ib/II trial (EPOC-2203) is now enrolling patients with HER2-low (IHC 2+/FISH—or IHC 1+) GEA, who will be treated with T-DXd, nivolumab and CAPOX, representing another interesting approach [124].

Thirty patients with HER2-positive mGEA, who had failed at least first-line treatment, were treated with disitamab vedotin + the anti-PD-1 mAb toripalimab in a Chinese phase-I trial [125]. ORR was 43%, median PFS 6.2 months (95% CI 4.0–6.9), and median OS 14 months (95% CI 6.3-NR). A multicenter real-world study concluded that ICI administration amplified the antitumor response of disitamab vedotin in HER2-positive and even HER2-low mGEA patients in third-line settings and beyond [126].

The aforementioned phase-Ib/II DESTINY-Gastric 03 trial investigates the combination of T-DXd with other biomarker-directed agents and consists of several substudies: Arm 1C receives treatment with T-DXd, the anti-PD-L1 mAb durvalumab and either capecitabine or 5-FU. The first results of this trial revealed promising antitumor activity and the manageable safety profile of T-DXd in combination with pembrolizumab ± CHT [127]. However, AE occurred more often than in patients treated with SOC (trastuzumab + platinum + FP) [127]. Particularly cohort D, which comprised 43 patients who received T-DXd 6.4 mg/kg + FP + pembrolizumab, had 39 (91%) AE of grade ≥ 3 [127]. The highest cORR was observed in patients receiving T-DXd + FP (78%, 32/41), while the second highest was achieved in the SOC treatment (76%, 22/29) [127]. Arms 3 and 4 will include the additional administration of bispecific antibodies volrustomig (PD-1 and cytotoxic T-lymphocyte associated protein 4 (CTLA-4)) and rilvegostomig (PD-1 and TIGIT (T-cell immunoglobulin and immunoreceptor tyrosine-based inhibitory motif domain)) [127].

The AIO INTEGA study investigated the efficacy of ipilimumab versus FOLFOX when combined with trastuzumab and nivolumab as first-line therapy in patients with ERBB2-positive GEA. Out of 88 randomized patients, the OS rate at 12 months was 57% (95% CI 41–71%) in the cohort treated with ipilimumab and 70% (95% CI 54–81%) in patients with FOLFOX [128]. The final survival results showed no superior PFS in the double ICI treatment arm either (3.2 vs. 10.7 months) [129]. While these data seem to favor the FOLFOX arm, the OS curves crossed with extended follow-up. Concluding median OS values were 22.1 and 23.3 months, leading to a numerical advantage for the ipilimumab cohort.

The administration of two bispecific antibodies, KN026 (targeting two distinct HER2 epitopes) and KN046 (blocking PD-L1 and CTLA4), was evaluated in a treatment-naive cohort of 31 patients with advanced HER2-positive GET [130]. Findings displayed notable efficacy, reliable safety, an ORR of 77.8%, and a DCR of 92.6% [130]. These promising results underscore the necessity of a large, randomized study to compare KN026 + KN046 versus the SOC.

Even though the synergy of combined anti-PD-1 and anti-HER2 therapy has already been demonstrated and research is progressing in this field, further randomized clinical trials are warranted to optimize therapy outcome. A summary on all clinical trials targeting both PD-(L)1 and HER2 in metastasized GET is provided within Table 1.

### 3.2. PD-(L)1 and CLDN18.2

Out of 311 assessed CLDN18.2 positive patients in the SPOTLIGHT trial, 41 (13%) had a PD-L1 CPS ≥ 5 [98]. This concurs with the results of a retrospective study, in which 21 (18%) of 117 tested CLDN18.2-positive tumor samples had a PD-L1 CPS ≥ 5 [100]. In the GLOW trial, the highest percentage of PD-L1 and CLDN18.2 positivity was observed (63/228 patients, 21.9%) [97]. When focusing on a lower cut-off (CPS ≥ 1), 69 out of 98 (74.2%) CLDN18.2 positive patients showed PD-L1 positivity in another retrospective analysis [96]. A third retrospective finding confirms again this observation in an Asian patient cohort, where 63 out of 80 (79%) CLDN18.2-positive (≥40%) GC patients showed a PD-L1 CPS ≥ 1 [132]. Clinical implications might be challenging, since both PD-L1 and CLDN18.2 inhibitors are approved options for those patients, with little evidence on the combination of those targets. Since there is an accordance of up to 80% of patients showing CLDN18.2 positivity and CPS ≥ 1, future trials focusing on combined treatment may provide a valuable benefit [102,132].

The third arm of the phase-II ILUSTRO trial covered three CLDN18.2 high or intermediate (CLDN18 staining is moderate to strong in 50%–75% of the tumor cells’ membranes) patient groups, who were treated with third-line or later zolbetuximab and pembrolizumab [133]. No partial or complete remission was observed and median PFS was 2.96 months (95% CI 1.44–2.48 months). As the very low number of patients and the late-line setting limit any meaningful clinical impact, a phase-II trial of first-line zolbetuximab in combination with nivolumab and mFOLFOX6 is currently recruiting patients with advanced or metastatic CLDN18.2-positive, HER2-negative gastric or gastroesophageal junction adenocarcinomas [134]. This single arm study will provide information on the tolerability of the combination of double target treatment. However, the small number of the study cohort will most probably be still not sufficient to provide enough data on the efficacy.

A novel mAb with improved affinity to CLDN18.2, osemitamab, displayed a synergistic interaction with anti-PD-1 mAbs and CHT in preclinical studies. In cohort G from the phase I/IIa TranStar102 study with 82 patients, osemitamab + nivolumab + CAPOX as first-line therapy showed encouraging antitumor activity, particularly in patients with medium/high CLDN18.2 activity (*n* = 32, median PFS 12.3 months, ORR 58.1%) [135].

Interestingly, the subgroup with PD-L1 CPS < 5 had even better results (*n* = 22, median PFS 12.6 months, ORR 71.4%) [135].

ASKB589 is a humanized IgG1 mAb targeting CLDN18.2 and is reported to have high affinity and enhanced antibody-dependent cellular cytotoxicity. In a phase-Ib/II trial, ASKB589 combined with CAPOX and the anti-PD-1 mAb sintilimab showed promising antitumor activity and manageable toxicity as first-line therapy [136]. Among the patient, 80% (12/15) achieved partial response, while the other 3 patients had stable disease, leading to an unconfirmed ORR of 80% and a DCR of 100%. Therefore, a subsequent phase-III trial was initiated in China to further explore these results in patients with CLDN18.2 positive, which is defined as ≥40% of tumor cells, advanced GET (NCT06206733) [137].

The phase-II GEMINI investigates the efficacy and safety of various novel ICI and CHT combination strategies in advanced untreated GEA patients [138]. In substudies 3 and 4, patients will be treated with the anti-CLDN18.2 mAb AZD0901 + FP + either volrustomig or rilvegostomig.

The bispecific antibody Q-1802 targets CLDN18.2 as well as PD-L1 and was administered to patients with GI tumors, including gastric adenocarcinoma in a single-arm phase-I trial [139]. In the dose-expansion cohort, 2 of 9 patients (22%) achieved partial response, and four exhibited stable disease (44%). With an excellent safety profile, Q-1802 showed preliminary antitumor activity and subsequent studies will follow. Now, there is a Chinese phase Ib/II trial ongoing, which investigates Q-1802 + XELOX vs. XELOX alone in untreated CLDN18.2 positive and HER-2 negative advanced GEA (NCT05964543) [139].

Several anti-CLDN18.2 strategies are now ongoing in different settings of GET, yet the current literature is not sufficient to provide a recommendation on the preference of biomarker-directed treatment in co-expressing patients.

In the case of CLDN18.2 co-expression in this patient cohort, the preference of anti-CLDN18.2 drugs over immunotherapy seems feasible. It is, however, a matter of debate, whether CPS levels could be further divided into subgroups based on the efficacy of ICI. Recently, outcome data were extracted from large clinical landmark trials including Checkmate-649, Keynote-859, and Rationale-305 and corresponding HR were provided [140]. According to this report, a moderate PD-L1 CPS expression level of 5–9 was linked to a reduced benefit from ICI, while patients with a PD-L1 CPS ≥10 experienced a significantly greater therapeutic benefit.

The threshold of CLDN18.2 positivity was selected after intensive research based on previous clinical studies [102,103]. However, it is not known if the efficacy of zolbetuximab further increases with elevated CLDN18.2 IHC levels. A recent report demonstrated the range of CLDN18.2 positivity obtained from SPOTLIGHT and GLOW trials and suggested a “very high” CLDN-18 positivity of 95–100% in 15% of patients [112]. Whether this was correlated with the outcome was not reported within this publication.

Table 2 shows all clinical trials involving combined CLDN18.2 and PD-1 treatment in metastasized GET.

### 3.3. PD-(L)1 and MSI

MSI-H is well known to be strongly associated with a high response rate to PD-(L)1 inhibitors, highlighting a significant correlation between PD-L1 expression and MSI [76]. In the KEYNOTE-062 trial, all 50/682 (7.4%) MSI-H patients had a PD-L1 CPS ≥ 1, 32 of whom had even a PD-L1 CPS ≥ 10 [76,82]. A similar powerful association was found in KEYNOTE-059 and -061 [76,80,81]. It is important to note that, in all of these three trials, PD-L1 expression was assessed using the same assay (PD-L1 IHC 22C3 pharmDx assay), so inter-assay discrepancy might be ruled out. In accordance with the previously described MSI-chapter, it is essential to point out the benefits MSI-H patients gain from immunotherapy. Pembrolizumab ± CHT provides significantly durable antitumor activity in GET patients regardless of the line of therapy in which it was received [76]. On the contrary, these findings are restricted since MSI-evaluation was not available in all patients, leading to a small number of patients and thus limited statistical interpretation.

However, a small proportion of MSI-H patients is not PD-L1 CPS-positive, leading to a hypothetical exclusion of CPS-based therapy regimens. Due to the inclusion criteria of KEYNOTE-061, all 50 MSI-H patients were therefore CPS-positive (CPS ≥ 1) too, whereas 11% (3/27) of those MSI-H patients in KEYNOTE-061 and 29% (2/7) in KEYNOTE-059 had CPS < 1 [76]. Kubota et al. observed that in 24 MMR-D patients, 8.3% had a CPS < 1 [96]. In order to provide this subgroup of patients with the best suitable therapy option, additional MSI testing is indispensable.

### 3.4. CLDN18.2 and HER2

To the best of our knowledge, there are scarce data on CLDN18.2 and HER2 interaction. A study focusing on the clinical and molecular characterization of CLDN18.2 found that, in 98 CLDN18.2 positive patients, 15 (15.3%) were HER2 positive [96]. 43 (13.9%) out of 310 CLDN18.2 negative patients showed HER2 positivity, indicating no significant correlation. Two further well-established cohorts also demonstrated HER2 positivity rates of 15% and 21% in CLDN18.2-positive patients [100,132]. Another research group similarly found no correlation between CLDN18.2- and HER2-expression [103]. The same study found that CLDN18.2 positivity was only predominantly observed in EBV-associated GC. To our knowledge, there is still no clinical trial planned to assess the combination of targeting both CLDN18.2 and HER2. However, this subgroup of patients (estimated ratio of approximately 4% in all stage IV GET) exerts a possibility to conduct research with bispecific antibodies or even bispecific antibody drug conjugates.

## 4. Biomarkers with Historical Background and High Potential for Future Implications

### 4.1. Vascular Endothelial Growth Factor (VEGF)

Targeting pro-angiogenic factors in order to decrease tumor supply with nutrients and thus tumor growth constitutes a vital component in various cancer therapies, including GC [20]. Particularly in second-line treatment of metastasized GC (mGC), ramucirumab, a mAb targeting vascular endothelial growth factor receptor-2 (VEGFR-2), plays a crucial role. The phase-III RAINBOW trial demonstrated the superiority of ramucirumab + paclitaxel vs. placebo + paclitaxel, leading to improved ORR, PFS, and OS in the ITT cohort (median OS 9.6 months vs. 7.4 months, HR 0.81, 95% CI 0.68–0.96, *p* = 0.017) [143,144]. Similarly, monotherapy with ramucirumab significantly prolonged OS and PFS, but not ORR, when compared to placebo/best supportive care in mGC [145]. Consequently, ramucirumab alone is recommended as a second-line treatment for mGC when there are contraindications for CHT [20]. In contrast, neither the addition of the anti-VEGF mAb bevacizumab (AVAGAST study) or ramucirumab (RAINFALL study) showed superior results when combined with fluoropyrimidine or platinum CHT in the first-line setting for mGC treatment [146,147]. Bevacizumab + CHT improved PFS and ORR, but not OS, whereas ramucirumab + CHT failed to improve both PFS and OS.

Since there are limited options after two prior therapy lines in GC, further clinical data are highly warranted. Therefore regorafenib, an oral multi-targeted tyrosine kinase inhibitor (TKI) targeting stromal, angiogenic and oncogenic receptor tyrosine kinases, was examined in mGC patients with at least two previous therapy lines. Superior OS was observed vs. best supportive care with a median of 4.5 vs. 4.0 months (HR 0.70, 95% CI 0.53–0.92, *p* = 0.011), whereas PFS was not significantly altered in patients with advanced GET [148].

Apatinib, also known as rivoceranib, a selective TKI of VEGFR-2, was examined in the phase-III ANGEL trial as a late-line strategy for advanced GET in a Western patient cohort [149]. When compared to best supportive care, rivoceranib improved PFS and ORR, but not OS, consequently failing the primary endpoint. However, in an all-Chinese population, rivoceranib led to significantly longer PFS and OS in patients with advanced GET in third-line and beyond, thus receiving approval of the China Food and Drug Administration in this setting [150]. This underlines the heterogeneity of GET across Western and Asian countries.

Fruquintinib, a highly selective small-molecule TKI inhibiting VEGFR-1, VEGFR-2, and VEGFR-3, combined with paclitaxel showed in a phase Ib study as second-line treatment for advanced GC promising results with a median PFS of 4 months and median OS of 8.5 months [151]. This led to the randomized phase III FRUTIGA trial, which examined the addition of fruquintinib + paclitaxel vs. paclitaxel alone in patients with mGEA who had progressed to first-line therapy. Median PFS was significantly longer in the ITT group (5.6 months) compared to the control group (2.7 months, HR 0.57, 95% CI 0.48–0.68, *p* < 0.001), while median OS was not significantly altered (9.6 vs. 8.4 months, HR 0.96, 95% CI 0.81–1.13, *p* = 0.606) [152].

The REGONIVO phase-Ib trial investigated the combination of regorafenib and nivolumab in 25 patients with advanced GET and at least two prior therapy lines [153]. Median PFS was 5.6 months and median one-year OS rate was 55.3%. Now there is an active phase-III trial, Integrate IIb, comparing regorafenib + nivolumab to SOC CHT in pretreated patients with advanced GET (NCT04879368), which already completed patient recruitment [154]. First-line regorafenib + nivolumab was also assessed in combination with CHT (FOLFOX) in advanced GEA [155]. The primary endpoint, 6-month PFS, was reached by 25/35 (71%) evaluable patients. Nine (26%) patients had a partial response and only one patient died, which was unrelated to treatment. A phase-III trial is planned.

Another phase-II study observed the favorable outcome of such combination therapy, i.e., lenvatinib (an oral multikinase inhibitor targeting VEGFR1-3 and other tyrosine kinases) and pembrolizumab as first- or second-line treatment in advanced GC patients. [156]. The primary endpoint was met, with an ORR of 69% in first- and second-line treatment of advanced GC patients, thus displaying promising antitumor activity.

Similarly to anti-VEGF(R) monotherapy/anti-VEGF(R) + CHT, this strategy of targeting PD-L1 and VEGFR has its limitations as first-line therapy but constitutes at the same time a valuable option for advanced GET with progressive disease.

Promising results were revealed by a Chinese phase II single-arm study investigating first-line tislelizumab + bevacizumab and CAPOX for mGEA with PD-L1 CPS < 5 [157]. DCR was 100% (27/27 patients) and ORR was 57.7%. Median PFS was 8.6 months (95% CI 6.8–10.4), meeting the primary endpoint of the study, which enables this regimen to be further explored (NCT05299476) [154]. Similar results were obtained from trials involving first-, second-line or maintenance therapy with camrelizumab (a PD-1 inhibitor), apatinib, and CHT in Chinese populations [158,159,160]. All of them showed favorable antitumor activity and a manageable safety profile.

Neuropilin-1 was found to stimulate the VEGF/VEGFR2 axis and has been identified as a potential biomarker in gastric cancer, as gastric cancer tissues seem to have a higher expression of this protein than normal gastric mucosa [161]. In a phase-II study with 35 mGEA patients, who were treated with CAPOX + bevacizumab, neuropilin-1 and -2 levels were associated with poorer survival [162]. Further research is needed to clarify the role of neuropilin-1 and -2 in GC and their impact on anti-VEGF therapy response.

In conclusion, targeting tumor angiogenesis and its most important molecular factor VEGF and receptor VEGFR plays a minor role in the first-line setting of advanced GET. Still, the combination of antiangiogenic and ICI treatment is currently being further explored, as promising results have already been revealed. In addition, ramucirumab represents a valuable option for patients who progressed under first-line therapy and do not have any contraindications for antiangiogenic therapy [20].

### 4.2. EGFR

EGFR alteration, i.e., amplification or overexpression, can be found in approximately 5–10% of GET (ESCAT II-B) [9,163,164]. Early on, promising preliminary results emerged indicating potential benefits from anti-EGFR therapy in mGEA patients regardless of EGFR status [165,166,167]. Supported by the generated data, three phase-III trials were designed to further explore this treatment strategy: REAL-3 (anti-EGFR mAb panitumumab + CHT, first-line), EXPAND (anti-EGFR mAb cetuximab + CHT, first-line), and COG (TKI gefinitib monotherapy, second-fourth line in esophageal AC and squamous cell cancer) [168,169,170]. All three studies failed to demonstrate beneficial results, which might be linked to patient selection, as inclusion was performed irrespective of EGFR status.

However, a biomarker analysis from the EXPAND trial concluded that there was a trend toward better OS, PFS, and ORR when cetuximab was combined with CHT in patients with high EGFR IHC scores, i.e., ≥10 [171]. In a post hoc analysis of the REAL-3 study, EGFR levels were assessed in plasma and tissue in the ITT population [172]. EGFR amplification in blood and tissue was found to be associated with poor survival. Interestingly, EGFR inhibition did not result in longer survival in patients with significantly elevated EGFR copy numbers [172]. To investigate this paradox, the interaction of anti-EGFR inhibitors and epirubicin, which was used as one CHT drug in the REAL-3 trial, was evaluated in patient-derived organoids from mGEA patients [172]. Adding anti-EGFR inhibitors to epirubicin led to the increased speed of cell cycle progression, consequently potentially antagonizing the antitumor effect of anthracyclines [172]. This shows the importance of the CHT backbone choice. The biomarker analysis of the COG trial discovered that patients with EGFR amplification (7.2%) had the biggest benefit from gefitinib treatment (HR for death 0.21, 95% CI 0.07–0.64, *p* = 0.006) [173]. A first approach of biomarker-selected anti-EGFR was seen in a prospective trial, in which 8/140 (6%) advanced GEA patients had EGFR amplification, and seven of them received at least one dose of anti-EGFR therapy. The ORR was 58% (4/7 patients), whereas the DCR was 100% [174].

Hence, the following data underline the relevance of patient selection in future prospective anti-EGFR trials, particularly focusing on tumors with EGFR-amplification in order to meet the highly warranted need in this patient cohort.

### 4.3. Mesenchymal–Epithelial Transition (MET)

The MET gene serves as an oncogenic driver with downstream effects contributing to mesenchymal–epithelial transition, tumor growth, and angiogenesis [175]. MET is part of the tyrosine kinase receptor group and together with its ligand hepatocyte growth factor (HGF), the HGF/MET pathway may act as a potential therapeutic target in GEA [176]. MET overexpression and amplification might suggest aggressive tumor characteristics and a less favorable clinical prognosis (ESCAT IIB for amplification and IIIA for mutation) [164,175]. The phase II VIKTORY umbrella trial aimed to examine genomic profiling-based treatment in mGC patients [177]. Among 715 patients, 20 (2.8%) had an MET-amplification and were treated with the MET-inhibitor savolitinib. Six-week PFS was 80% and an ORR was observed in 10/20 (50%) patients, which was the best ORR among the 10 biomarker-based treatment arms.

The phase-III RILOMET-1 investigated the addition of the mAb targeting HGF, namely rilotumumab, to CHT in advanced MET-expressing mGEA with no previous treatment [178]. Rilotumumab failed to improve clinical outcomes, with a median OS of 8.8 months (95% CI 7.7–10.2) in the ITT group vs. 10.7 months (95% CI 9.6–12.4) in the placebo group (stratified HR 1.34, 95% CI 1.10–1.63, *p* = 0.003). RILOMET-1 and the following RILOMET-2 study had to be terminated as a result of rising deaths in the ITT group [179]. Similar results were obtained from another phase-II trial examining the efficacy of the anti-MET mAb onartuzumab + mFOLFOX6 in an unselected metastatic HER2-negative GEA population [180]. In the MET-positive patient cohort, median OS was 8.51 months in the onartuzumab group vs. 8.48 months in the placebo group (HR, 1.12, 95% CI 0.45–2.78, *p* = 0.80) [180].

Various other MET-inhibitors were also examined in clinical trials: Foretinib, an oral multikinase inhibitor including MET and VEGFR2 did not show any improvements in an unselected patient cohort of 74 mGC patients [181]. In an Asian mGC cohort of pretreated 30 patients, monotherapy with the selective MET-inhibitor tivatinib showed only limited efficacy, with no ORR observed [182]. ABBV-400, a novel a-Met protein-targeting ADC (mAb telisotuzumab conjugated to a novel toposiomerasae 1 inhibitor payload), was observed to display a tolerable safety profile combined with antitumor activity in 42 pretreated patients with mGEA [183]. In this phase I study, ORR was 28.6% and median PFS 4.0 months (95% CI 2.8–5.0 months).

Amivantamab, a novel bispecific antibody targeting both EGFR and the MET receptor, was assessed as late-line therapy in 29 GEA patients [184]. The primary endpoint (ORR ≥ 20%) was not met and GEA in particular experienced less benefits than patients with esophageal squamous cell cancer.

Overall, while MET remains a promising therapeutic target in GEA, the limited success of MET inhibitors in clinical trials potentially suggests a need for more personalized and differentiated treatment approaches.

### 4.4. EBV

As one of the four molecular subtypes according to the TCGA, EBV is found in approximately 8–9% of all GCs [9,185]. It is associated with PD-L1 and PD-L2 overexpression, which might consequently influence ICI prognosis. A study focusing on a next-generation-sequencing-based model concluded that EBV positivity was similarly effective to MSI in the prediction of immunotherapy outcome in GC [186]. Impressively, 100% (6/6) of Korean patients with EBV-positive metastatic GC had an ORR, i.e., at least partial response with pembrolizumab as later line treatment [187]. However, late-stage EBV-associated GC accounts for only 3% of all cases, which limits the potential sample size, as one study discussed after examining nine advanced EBV-positive patients with ICI therapy who showed favorable—but not as good as expected—outcomes [188]. In an exploratory analysis of six CHT-refractory patients with advanced or metastatic EBV-encoded RNA positive GC, camrelizumab did not lead to any ORR, which led to the termination of the phase-II study [189]. Aside from the Korean patient cohort with a remarkable response rate, different studies did not confirm these results, as the ORR did not exceed 28.6% in any one of those [187,188,189,190,191].

Therefore, the impact of immunotherapy in EBV-positive GC remains ambivalent and requires further insight. Currently, there are several ongoing clinical trials recruiting EBV-positive non-metastatic patients aiming to display the significance of EBV for immunotherapy in GET [192,193].

## 5. Future Biomarkers

### 5.1. Fibroblast Growth Factor Receptor (FGFR)

The fibroblast growth factor family (FGF) and its receptor FGFR are transmembrane growth factor receptors present in numerous tissues. The FGF/FGFR signaling pathway plays a crucial role in neoplastic activity as well as in development, differentiation, growth, and survival of cells [194]. Particularly, the amplification of FGFR2 was found to be specific for GC [9]. The occurrence of FGFR2b overexpression (moderate to strong in at least 5% of cells) is about 30% in HER2-negative patients, whereas amplification of FGFR is only seen in about 4–9% of GC patients [9,194,195]. In terms of biomarker co-expression, there is limited overlap with other targetable biomarkers such as PD-L1 or CLDN18.2, which was revealed in a Japanese cohort with 130 patients [196]. An irreversible FGFR1–4 inhibitor, futibatinib, was examined in several solid tumors including GC. In two phase-I trials, futibatinib demonstrated preliminary efficacy with acceptable safety, leading to the initiation of a phase-II trial [197,198].

Bemarituzumab, an afucosylated mAb against FGFR2b, induced confirmed objective responses in 5 (18%) out of 28 patients with CHT-refractory gastric adenocarcinoma characterized by FGFR2 amplification and elevated FGFR2b overexpression [199]. In the following phase-II FIGHT trial, bemarituzumab was non-superior to placebo regarding the outcome of PFS in patients with FGFR2b-overexpression advanced GEA [195]. Median PFS was 9.5 months in the bemarituzumab + mFOLFOX6 group vs. 7.4 months in the placebo + mFOLFOX6 group (HR 0.68, 95% CI 0.44–1.04, *p* = 0.073) [195]. A survival benefit was also observed, with a median OS of 19.2 months (95% CI 13.6—not reached) in the bemarituzumab group vs. 13.5 months (95% CI 9.3–15.9) in the placebo group (HR 0.60, 95% CI 0.38–0.94). However, stomatitis and corneal adverse events were frequently observed, which are well-known side effects of inhibiting the FGF-FGFR pathway. To understand the effects and the benefit of bemarituzumab better, two phase-III trials were initiated in untreated GET patients with FGFR2b overexpression: FORTITUDE-101 compares bemarituzumab + mFOLFOX6 vs. placebo + mFOLFOX6, whereas FORTITUDE-102 investigates the addition of bemarituzumab to nivolumab + CHT vs. placebo + nivolumab + CHT [200,201].

### 5.2. T-Cell Immunoglobulin and Immunoreceptor Tyrosine-Based Inhibitory Motif Domain (TIGIT)

TIGIT, a surface molecule on T-cells, acts as an immune checkpoint that suppresses T-cell responses and significantly contributes to the development and progression of GC [202]. Research indicates that GC patients had an elevated proportion of TIGIT+ CD8 T-cells compared to healthy individuals, suggesting that TIGIT might be a potential prognostic biomarker in GC [202,203]. Dual ICI combination with PD-1 inhibition has been recommended as a strategy to address this patient cohort [204].

Consequently, patients with locally advanced unresectable or mGEA were experimentally treated with domvanalimab, an anti-TIGIT Fc-silent mAb, zimberelimab, an anti-PD-1 mAb and FOLFOX in the phase-II EDGE trial (*n* = 41) [205]. Adding domvanalimab and zimberelimab to FOLFOX led to 59% ORR and a 6-month PFS rate of 75% [205]. Especially PD-L1-high patients (TAP ≥ 5, *n* = 14) had the greatest benefit, with an ORR of 80% and 6-month PFS rate of 93% [205].

A different TIGIT inhibitor, ociperlimab, exhibited encouraging antitumor effects in patients with metastasized GET [206].

Rilvegostomig represents a novel bispecific mAb targeting both PD-1 and TIGIT, which was currently investigated in substudy 2 of the ongoing multicenter GEMINI-Gastric phase-II trial (NCT05702229). The first report demonstrated a favorable outcome of rilvegostomig + CHT and a manageable safety profile in 40 patients with HER2-negative mGEA, leading to a confirmed ORR of 52.5% and a DCR of 100% [141].

Although these trials provided promising results on the efficacy of anti-TIGIT inhibitors, the real benefit of these compound on top of anti-PD(L)-1 inhibition is still missing. This question will be examined in the phase-III STAR-221 trial, where double ICI + CHT will be tested against PD-1 inhibition together with chemotherapy [207]. This trial is still recruiting, and results are yet to be revealed.

Further clinical trials are warranted to solidify the position of TIGIT and its targeted therapy in GET patients.

### 5.3. Dickkopf-1 (DKK-1)

DKK1, known as a modulator of Wnt signaling, is frequently overexpressed in various types of cancer and often tied to poor clinical outcomes due to immunosuppressive effects [208]. In a murine model, treatment with the anti-DKK1 mAb DKN-01 was found to stimulate innate immunity responses in the tumor microenvironment and had synergistic effects with PD-1 inhibitors [208]. Similar results were obtained from a phase-I trial in advanced GET, in which DKN-01 was combined with pembrolizumab [209]. Augmented antitumor response was observed in patients with a high DKK-1 expression and no prior anti-PD-1/-L1 therapy.

The administration of first-line DKN-01 together with the anti-PD1 inhibitor tislelizumab and CAPOX was investigated in advanced GEA patients (*n* = 25) in the phase-II DisTinGuish trial [210]. The results of the 2-year follow-up revealed a median PFS of 11.3 months in the overall ITT cohort and 10.3 months in the low PD-L1 group (CPS < 5, *n* = 18) [211]. These survival data are superior to those of historical SOC therapy of nivolumab + CHT reports, in the overall population (median PFS 11.3 vs. 7.7 months; median OS 19.5 vs. 13.8 months), as well as in the low PD-L1 group (median PFS 10.7 vs. 7.5 months; median OS 18.7 vs. 12.4 months) [211]. Part C of this trial investigates whether the addition of DKN-01 to tislelizumab and CHT is beneficial as first-line treatment in GEA patients, with no published data to date. The combination of those three discussed future biomarkers with PD-1 is shown in Table 3.

## 6. Discussion

Despite considerable progress in the management of GC, ongoing basic research as well as clinical trials are desperately warranted. The primary goals remain the improvement of early detection, minimizing relapse, and enhancing therapeutic approaches. Moreover, the need for clinically feasible biomarkers has to be thoroughly addressed, particularly the co-expression of such. With the initiation of combined targeted therapies, substantial advances have been made in GET [118], and yet newly discovered targets have to be examined together with already approved ones.

There is almost worldwide consensus on the determination of specific biomarkers prior to systemic treatment of metastatic GET. Although PD-L1 assessment using CPS is standard, various positivity thresholds have been associated with the approval of different ICI drug agents [20]. One explanation for this discrepancy is the different design of clinical trials with differing statistical assumptions and power. Another hypothesis is that response to immunotherapy might vary with CPS expression levels. European GC and GEJ guidelines recommend nivolumab + chemotherapy in patients with a CPS ≥ 5, whereas American NCCN guidelines state that this regimen may be also useful under certain circumstances in patients with CPS < 5 [20,55]. Similarly, ESMO living guidelines state that, for patients with a CPS 1–4, ICI may be considered [212]. The same difficult situation becomes apparent when considering the administration of pembrolizumab and chemotherapy as a first-line therapy in patients with esophageal or gastroesophageal junction adenocarcinoma. The official approval requires a CPS value of ≥ 10, and yet a less strong recommendation may be found for lower CPS-levels [55]. As our understanding of the therapeutic value of CPS as a biomarker increases and new clinical trials emerge, the consideration of the extent of CPS expression will become more important in decision making.

A typical example in clinical routine is dual biomarker expression patterns. PD-L1 and CLDN18.2 co-expression occurs in a significant number of patients and several studies addressing the dual inhibition of these markers are currently ongoing [134,135,136,138,139]. From today’s point of view, a critical patient selection on the basis of the extent of expression of an individual biomarker seems to be crucial. Patients with a low CPS expression are expected to gain less benefit from immunotherapy according to the guidelines [20,55], so in case of co-expression with CLDN18.2, a targeted therapy with zolbetuximab might be preferred. It may be argued that ICI administration might be favored over zolbetuximab in patients exhibiting higher CPS values or other strong immune markers, including MSI-H, TMB-H, and EBV, if CLDN18.2 expression co-occurs. Another criterion for patient selection is the tolerability profile. Patients with organ transplantations or autoimmune diseases diagnosed with metastatic GET are often excluded from clinical trials. Therefore, the application of immunotherapy is still controversial for those patients, even if CPS is highly expressed. Targeted therapy might serve as an additional option in those patients. As previously described, there is still no clinical trial aimed at targeting both CLDN18.2 and HER2, which is observed in a small but significant subgroup of patients with metastatic GET [100,132]. In line with this, personalized medicine according to the expression of individual biomarkers is highly warranted and requires further investigation.

As in all medical fields, the use of AI is being explored, which might play a future crucial role in predicting treatment responses. Besides the already mentioned AI-based single-cell computational pathology strategy, an AI-powered analyzer was shown to assess the immune phenotype and be capable of predicting outcomes in patients with immunotherapy [213]. Patients with an inflamed immune phenotype had significantly better survival rates. A multi-modal deep learning model integrating clinical, radiological, and pathological data was already able to accurately predict treatment response in HER2-positive GC [214]. AI divided patients into low-risk and high-risk groups, which then correlated with OS and PFS. Data incompleteness was addressed through learnable modality-specific embeddings acting as placeholders for missing data. Particularly, the inclusion of multi-modal datasets adds to the pursuit of personalized treatment. However, the full impact of AI in cancer treatment is yet to be unveiled. Results of the aforementioned approaches underscore the potential applicability of AI models in future clinical routine [71,213,214].

At the moment, various novel ICIs are being examined in the already mentioned large-phase-II GEMINI-Gastric trial assigning 240 advanced or metastasized GEA patients across six substudies (NCT05702229) [138]. Among those novel drugs, the focus shifts to dual biomarker inhibition, i.e., bispecific antibodies targeting PD-1 and CTLA4, or PD-1 and TIGIT. Nonetheless, it is essential to display that dual ICI inhibition once failed compared to CHT alone. Nivolumab and ipilimumab were inferior to CHT in terms of PFS and OS in the previously mentioned Checkmate 649 trial [215]. These findings do not prevent research from shifting focus from single immunotherapy to combining agents with different mechanisms of action in trials targeting multiple pathways, with the aim of overcoming resistance mechanisms and achieving higher anti-PD1/PD-L1 efficacy [216]. To reach this aim, diverse components accounting for the tumor as a whole have to be fully explored. Such components are epigenetics, immunosuppression, microbiota, tumor metabolism, and of course, biomarker expression [8]. Once the underlying mechanisms of resistance, immune evasion, and biomarker interaction are fully understood, treatment strategies may become more efficient. These treatment strategies also include later-line settings, as progression under first-line therapy is only a question of time. Therefore, a significant part of all resources is also directed towards second-line treatment options and beyond for GET. So, innovative personalized treatment options are on the rise and the horizon of various anticancer drugs is expanding.

An optimized fusion of all well-known therapy options including the older (CHT and radiotherapy) and the recent (ICI and biomarker-directed) ones is currently the trend towards the future. As described in this review, the focus might shift towards the interaction of expressed biomarkers by tumors, so that each patient can benefit from a specific personalized treatment.

## 7. Conclusions

Novel systemic treatment strategies, aiming to improve the outcomes of patients with metastasized GET, are underway. These approaches are based on various molecular biomarkers, which are the basis for personalized medicine, as each individual patient shows different (co-)expression clusters. Although it seems impossible to examine every combination of targeted agents in clinical trials, more and more advances have been made to reach this goal. Particularly, the comparison of newly discovered therapy regimens to standard of care treatment will be crucial in the future. While groundbreaking new drugs have significantly advanced GC treatment, extensive clinical research is still essential to push precision medicine further forward. As physicians are faced with a broad variety of therapeutic options including immune checkpoint inhibitors, targeted therapies, established chemotherapy regimens, and ADCs, it is now challenging to select the optimal approach to pursue the greatest benefit for patients. An enhanced understanding of biomarker expression patterns will guide physicians to choose the best suitable biomarker-based treatment option in clinical routine.

## Figures and Tables

**Table 1:** Clinical trials focusing on anti-HER2 and -PD-(L)1 co-expression treatment in metastasized GET.

Name	National Clinical Trial Number	Phase	N	Target	Line	Treatment	Primary Endpoint	Recruitment Status	Outcome	References
KEYNOTE-811	NCT03615326	III	698	HER2 + PD-1	1st line	Trastuzumab + FP + platinum-based CHT + pembrolizumab vs. placebo	PFS, OS	Active, not recruiting (international)	mPFS 10.0 vs. 8.1 mo mOS 20.0 vs. 16.8 mo	[118,120]
MAHOGANY Cohort A	NCT04082364	II/III	43	HER2 + PD-1	1st line	Margetuximab + retifanlimab	ORR	Active, not recruiting (international)	ORR 53% DOR 10.3 mo	[122]
HERIZON-GEA-01 Arm C	NCT05152147	III	918 *	HER2 + PD-1	1st line	Zanidatamab + tislelizumab + CAPOX or FP	PFS, OS	Active, recruiting (international)	Ongoing	[123]
EPOC-2203	jRCT2031230477	Ib/II	31	HER2 + PD-1	1st line	T-DXd + nivolumab + CAPOX	DLT (Ib), ORR (II)	Active, recruiting (Japan)	Ongoing	[124]
/	NCT04280341	I	56	HER2 + PD-1	≥2nd line	Disitamab vedotin + toripalimab	DLT, MTD, TRAE	Active, recruiting (China)	^1^ cORR 43% mPFS 6.2 mo mOS 16.8 mo	[125]
DESTINY-Gastric03 Arm 1C and E	NCT04379596	Ib/II	413 *	HER2 + PD- L-1	1st line	T-DXd + durvalumab ± 5-FU	Safety, INV cORR	Active, recruiting (international)	Ongoing	[127,131]
Arm 2D		II	43	HER2 + PD-1	1st line	T-DXd + pembrolizumab + FP	Safety, INV cORR	Active, not recruiting (international)	cORR 58%	
Arm 2E		II	41	HER2 + PD-1	1st line	T-DXd + pembrolizumab	Safety, INV cORR	Active, not recruiting (international)	cORR 66%	
Arm 3		II	n.a	HER2 + PD-1 +CTLA4	1st line	T-DXd + Volrustomig + FP	Safety, INV cORR	Active, recruiting (international)	Ongoing	
Arm 4		II	n.a	HER2 + PD-1 +TIGIT	1st line	T-DXd + Rilvegostomig + FP	Safety, INV cORR	Active, recruiting (international)	Ongoing	
INTEGA	NCT03409848	II	97	HER2 + PD-1 + CTLA4	1st line	Trastuzumab + nivolumab + ipilimumab vs. FOLFOX	OS rate at 12 mo	Completed (Germany)	^2^ 12 mo OS: 57% vs. 70% mPFS 3.2 vs. 10.7 mo ORR 34% vs. 53.5%	[128,129]
KN026-203	NCT04521179	II	31	HER2 + PD1 +CTLA4	1st line	KN026 + KN046	ORR, DOR	Completed	^3^ ORR77.8% DCR 92.6%	[130]

* estimated enrollment of whole trial; ^1^: out of 30 patients with gastroesophageal tumors; ^2^: out of 88 randomized patients; ^3^: 27 patients were evaluable for efficacy assessment. Abbreviations: CAPOX = capecitabine + oxaliplatin; CHT = chemotherapy; (c)ORR = (confirmed) overall response rate; CTLA4 = cytotoxic T-lymphocyte-associated protein 4; DCR = disease control rate; DLT = dose-limiting toxicity; DOR = duration of response; FOLFOX = folinic acid (leucovorin) + 5-fluouracil + oxaliplatin; FP = fluoropyrimidine (5-fluorouracil or capecitabine); HER2 = human epidermal growth factor receptor 2; INV = investigator-assessed; (m)OS = (median) overall survival; (m)PFS = median progression-free survival; mo = months; MTD = maximum-tolerated dose; N = number of patients; n.a = not available; PD-1 = programmed death receptor 1; T-DXd = trastuzumab–deruxtecan; TRAE = treatment-related adverse event.

**Table 2:** Clinical trials focusing on anti-CLDN18.2 and PD-(L)1 co-expression treatment in metastasized GET.

Name	National Clinical Trial Number	Phase	N	Target	Line	Treatment	Primary Endpoint	Recruitment Status	Outcome	References
ILUSTRO Arm 3 Arm 4	NCT03505320	II	3 ≅50	CLDN18.2 + PD-1	3rd line or later 1st Line	Zolbetuximab + pembrolizumab Zolbetuximab +nivolumab + FOLFOX	ORR	Active, not recruiting (international) Active, recruiting (international)	ORR 0% DCR 66.7% mPFS 2.96 mo ongoing	[133,134]
TranStar102 Cohort G Cohort H	NCT04495296	I/IIa	82 n.a	CLDN18.2 + PD-1 CLDN18.2 + PD-1	1st line 1st line	Osemitamab + nivolumab + CAPOX Osemitamab + nivolumab	TRAE, DLT, MTD, RP2D	Active, recruiting (China)	^3^ mPFS 12.3 mo ORR 58.1% ongoing	[135]
/	NCT05632939	Ib/II	26	CLDN18.2 + PD-1	1st line	ASKB589 + sintilimab + CAPOX	TRAE, DLT, MTD, RP2D	Active, recruiting (China)	^4^ ORR 80% 100% DCR, PR 80%	[136]
GEMINI-Gastric Substudy 3	NCT05702229	II	≅40	CLDN18.2 + PD-1+CTLA4	1st line	AZD0901 + volrustomig + FP	ORR, PFS	Active, recruiting (International)	Ongoing	[141]
Substudy 4			≅40	CLDN18.2 + PD-1+TIGIT		AZD0901 + rilvegostomig + FP			Ongoing	
/	NCT05964543	Ib/II	72 *	CLDN18.2 + PD-L1	1st line	Q-1802	TRAE, ORR	Active, recruiting (China)	Ongoing	[142]

* estimated enrollment of whole trial; ^3^: median follow-up at 12.6 months, results for medium/high CLDN18.2 subgroup (*n* = 32); ^4^: For 15 evaluable patients who had at least one post-baseline tumor assessment. Abbreviations: CAPOX = capecitabine + oxaliplatin; CLDN18.2 = claudin 18.2; (c)ORR = (confirmed) overall response rate; CTLA4 = cytotoxic T-lymphocyte-associated protein 4; DCR = disease control rate; DLT = dose-limiting toxicity; DOR = duration of response; FOLFOX = folinic acid (leucovorin) + 5-fluouracil + oxaliplatin; FP = fluoropyrimidine (5-fluorouracil or capecitabine); INV = investigator-assessed; (m)OS = (median) overall survival; (m)PFS = median progression-free survival; MTD = maximum-tolerated dose; N = number of patients; n.a = not available; ORR = overall response rate; PD-1 = programmed death receptor 1; RP2D = recommended phase 2 dose; T-DXd = trastuzumab–Deruxtecan; TIGIT = T-cell immunoglobulin and immunoreceptor tyrosine-based inhibitory motif domain; TRAE = treatment-related adverse event.

**Table 3:** Summary of clinical trials focusing on PD-1 and future biomarker co-expression.

Name	National Clinical Trial Number	Phase	N	Target	Line	Treatment	Primary Endpoint	Recruitment Status	Outcome	Reference
FORTITUDE-102	NCT05111626	Ib/III	528	FGFR2b + PD1	1st line	Bemarituzumab + nivolumab + CAPOX or mFOLFOX6 vs. placebo + nivolumab + CAPOX or mFOLFOX6	Part 1: DLT, TRAE Part 2: OS	Active, recruiting (international)	Ongoing	[201]
EDGE	NCT05329766	II	41	TIGIT + PD-1	1st line	Domvanalimab + zimberelimab + FOLFOX	Safety, ORR	Active, recruiting (international)	ORR 59% 6-month PFS rate 75%	[205]
AdvanTIG-105	NCT04047862	Ib	60	TIGIT + PD-1	1st line	Ociperlimab + tislelizumab + CAPOX/FP	ORR	Active, not recruiting (international)	ORR 50.8% mPFS 8.2-month DCR 84.7%	[206]
STAR-221	NCT05568095	III	1040 *	TIGIT + PD-1	1st line	Domvanalimab + zimberelimab + FOLFOX/CAPOX vs. placebo + nivolumab + FOLFOX/CAPOX	OS	Active, not recruiting (international)	Ongoing	[207]
DisTinGuish	NCT04363801	II	232 *	DKK-01 and PD-1	1st line	DKN-01 + tislelizumab + CAPOX or mFOLFOX6	PFS	Active, not recruiting (international)	Ongoing	[211]

* estimated enrollment of whole trial; Abbreviations: CAPOX = capecitabine + oxaliplatin; (c)ORR = (confirmed) overall response rate; DCR = disease control rate; DKK-01 = Dickkopf-01; DLT = dose-limiting toxicity; FGFR2b = fibroblast growth factor receptor 2b; FOLFOX = folinic acid (leucovorin) + 5-fluouracil + oxaliplatin; FP = fluoropyrimidine (5-fluorouracil or capecitabine); INV = investigator-assessed; (m)OS = (median) overall survival; (m)PFS = median progression-free survival; N = number of patients; PD-1 = programmed-death receptor 1; TIGIT = T-cell immunoglobulin and immunoreceptor tyrosine-based inhibitory motif domain; TRAE = treatment-related adverse event.
